# Total and Free Blood and Plasma Concentration Changes in Pregnancy for Medicines Highly Bound to Plasma Proteins: Application of Physiologically Based Pharmacokinetic Modelling to Understand the Impact on Efficacy

**DOI:** 10.3390/pharmaceutics15102455

**Published:** 2023-10-13

**Authors:** Paola Coppola, Andrew Butler, Susan Cole, Essam Kerwash

**Affiliations:** Medicines and Healthcare Products Regulatory Agency (MHRA), 10 South Colonnade, London E14 4PU, UK; andrew.butler@mhra.gov.uk (A.B.); susan.cole@mhra.gov.uk (S.C.); essam.kerwash@mhra.gov.uk (E.K.)

**Keywords:** pharmacokinetics, pregnancy, protein binding, free concentrations, total concentrations, PBPK, regulatory

## Abstract

Free drug concentrations are generally considered the pharmacologically active moiety and are important for cellular diffusion and distribution. Pregnancy-related changes in plasma protein binding and blood partitioning are due to decreases in plasma albumin, alpha-1-acid glycoprotein, and haematocrit; this may lead to increased free concentrations, tissue distribution, and clearance during pregnancy. In this paper we highlight the importance and challenges of considering changes in total and free concentrations during pregnancy. For medicines highly bound to plasma proteins, such as tacrolimus, efavirenz, clindamycin, phenytoin, and carbamazepine, differential changes in concentrations of free drug during pregnancy may be clinically significant and have important implications for dose adjustment. Therapeutic drug monitoring usually relies on the measurement of total concentrations; this can result in dose adjustments that are not necessary when changes in free concentrations are considered. We explore the potential of physiologically based pharmacokinetic (PBPK) models to support the understanding of the changes in plasma proteins binding, using tacrolimus and efavirenz as example drug models. The exposure to either drug was predicted to be reduced during pregnancy; however, the decrease in the exposure to the total tacrolimus and efavirenz were significantly larger than the reduction in the exposure to the free drug. These data show that PBPK modelling can support the impact of the changes in plasma protein binding and may be used for the simulation of free concentrations in pregnancy to support dosing decisions.

## 1. Introduction

**Figure 1:**
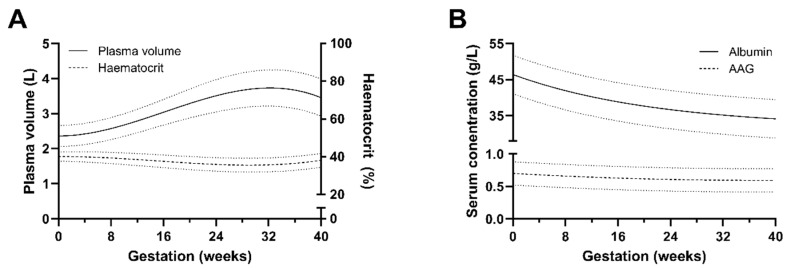
(**A**) Changes in plasma volume (left axis) and blood haematocrit (right axis) during pregnancy. (**B**) Changes in the serum plasma proteins—albumin and alpha-1-acid glycoprotein—during pregnancy. Dotted lines in each represent standard deviations. All the values are calculated using the formulae presented in Dallmann et al., in 2017 [5].

Pregnancy-related physiological changes may affect the pharmacokinetics of medicines, leading to possible alterations in absorption, distribution, metabolism, and excretion processes. Changes in plasma proteins’ concentrations, glomerular filtration rate (GFR), hepatic blood flow, metabolic enzymes, and transporters’ activity may influence the systemic exposure and elimination pathways of several drugs that are cleared either via the kidney or liver [1,2]. As a consequence of the altered activity of metabolism enzymes, the blood concentrations of drugs metabolised through CYP2D6, CYP3A4, CYP2A6, UGT1A4, and UGT2B7 are expected to decrease during pregnancy compared to nonpregnant subjects, while the concentrations of drugs that are substrates of other enzymes, e.g., CYP1A2 and CYP2C19, are expected to increase in pregnant women [3]. Although changes in some P450 enzymes have been well characterised and reviewed [3], changes in UGTs and transporters are less well understood; however, some changes are documented and have been recently reviewed [4]. Plasma protein binding and blood partitioning of drugs decrease during pregnancy due to changes in blood volume, reduced haematocrit, and reduced concentrations of both albumin and alpha-1-acid glycoprotein (AAG). This would lead to higher concentrations of free drug and increased tissue distribution and clearance. Haematocrit values decrease over pregnancy with an estimated 5% decrease up to 31 weeks, and there have been some reports of a slight return and increase towards the end of pregnancy [5]. The estimated mean albumin concentrations gradually decrease during pregnancy, whereas concentrations rapidly increase postpartum and up to 15 weeks after delivery. This may be related in part to increased plasma volume and increased urinary albumin excretion. The estimated AAG concentrations remain relatively stable and only slightly decrease from 24 weeks of gestation to term (Figure 1).

The free fraction represents the fraction of the drug in the plasma that is unbound to the plasma proteins and is generally considered to be the pharmacologically active moiety. Changes in plasma constituents and, hence, binding have been noted in a number of special populations, including pregnancy [6]. This can result in differential changes between the total and free drug in the population, with important implications for the understanding of the efficacy of the drug in question, at a given dose, in the population. For a number of agents (e.g., carbamazepine, phenytoin, valproate acid, tacrolimus, lopinavir, efavirenz, clindamycin, cefazolin, prednisone, and levothyroxine), an increased fraction that is unbound during pregnancy has been discussed in the literature [7,8,9,10,11,12]. Several of these drugs show a decreased exposure in pregnancy (e.g., tacrolimus and efavirenz), in some cases leading to dose increases [7,13,14]. However, despite the observed decrease in the total concentrations, dose adjustment might not be needed in pregnancy if the reduced total exposure is not associated with a proportional decrease in the free concentrations, e.g., lopinavir [9] and tacrolimus [7]. This highlights the importance of understanding the exposure in terms of free concentrations in pregnancy.

In some cases, therapeutic drug monitoring is recommended to monitor the exposure and dose adjustments recommended based on these levels (e.g., tacrolimus and phenytoin [15,16]). The measured parameter is usually the total drug and the considerations on dosing adjustments rely on total concentrations only with the aim of achieving total, systemic drug concentrations in pregnancy that are comparable to those in nonpregnant subjects. Ideally, free concentrations should be measured; however, the measurement of these concentrations is challenging as it requires reliable methods for separating the total and free drug fractions and highly sensitive methods for detecting the small quantities of unbound drug. As such, therapeutic drug monitoring during pregnancy may not correlate with changes in a pharmacologically active free drug. In addition, even though drug measurement of the total or free drug may be possible, the turn-around time for the bioanalysis and the clinician’s decision can be in the range of two weeks, and changes in exposure in pregnancy are known to occur very quickly, resulting in a delay in optimised treatment.

The physiological changes occurring in pregnancy are mechanistically incorporated into a number of pregnancy physiologically based pharmacokinetics (PBPK) system models that are publicly available: PKSim (http://www.open-systems-pharmacology.org/, SIMCYP Simulator (Certara UK Ltd., Sheffield, UK) and Gastroplus (SimulationsPlus Inc., Lancaster, CA, USA). During the development of these models, an extensive collection of the literature’s information was performed [5,17]. Changes in blood volume, haematocrit, and the predominant plasma proteins—albumin and AAG—along with other physiological parameters, are included in the models as described by a number of quadratic equations. A graphical example of the resulting equations used in PKSim is shown in Figure 1. The binding of the drug of interest to the blood cells and the predominant plasma proteins is described in the drug model either as a ratio, fraction unbound or, mechanistically, as dissociation constants, Kds. Consequently, these PBPK models could be used to predict changes in exposure, as plasma protein concentrations change, in the pregnant population of both free and total drug and, possibly, to support dosing strategies [18,19].

In this paper, we highlight the importance of considering changes in total and free concentrations in pregnancy and explore the potential of the PBPK models to support the understanding of the changes in drugs binding to plasma proteins and for the simulation of free concentrations in pregnancy. Tacrolimus and efavirenz, both highly bound to plasma proteins, are used as two example drug models. Pregnancy PBPK modelling was used for both drug models to simulate the total and free drug fraction in the plasma and blood of the virtual pregnant population.

## 2. Efavirenz

Efavirenz is indicated in the antiviral combination treatment of human immunodeficiency virus-1 (HIV-1)-infected adults, adolescents, and children 3 months of age and older, weighing at least 3.5 kg [20].

The Efavirenz’s summary of product characteristics (SmPC) states that this medicine should not be used in pregnant women unless the patient’s clinical condition requires such treatment [20]. However, there is evidence of the use of efavirenz during pregnancy [20,21,22]. Efavirenz is reported to be highly bound (approximately 99.5–99.75%) to human plasma proteins (predominantly albumin), and it is mainly metabolised by CYP3A4 and CYP2B6 [20]. As CYP2B6 is a polymorphic enzyme, increased PK variability may be observed in the pregnant population due to the increased activity of this enzyme during pregnancy.

A slight decrease in efavirenz’s systemic total exposure was observed between pregnancy (second and third trimester) and postpartum after treatment with 600 mg of efavirenz once daily [23,24]. Lartey et al. [24] observed a 16, 12, and 10% decrease in efavirenz’s AUC, Cmin, and Cmax, respectively, in pregnancy compared to postpartum, as well as a 20% increase in CL/F. Similarly, Kreimann et al. [23] and Cressey et al. [25] show up to a 25% decrease in AUC, Ctrough, and Cmax in pregnancy compared to postpartum, but a lower difference was observed when comparing the third trimester and postpartum. The authors suggested that the magnitude of the observed differences was sufficient to warrant a dosing adjustment [23,24,25].

A non-inferior efficacy was observed when the dose of efavirenz was reduced from 600 to 400 mg in the third trimester; however, the PK parameters, AUC0-24 h, and plasma concentrations 24 h post-dose were slightly lower in the third trimester than during postpartum, even though they remained within the therapeutic range [26,27,28]. Kreitchmann reported that C24 (24 h post-dose) concentrations during the second and third trimester were similar to the C24 concentrations seen in the ENCORE1 study with a 400 mg dose [23,27]. However, the increased variability due to CYP2B6’s polymorphism could lead to the subtherapeutic, systemic exposure in extensive metabolisers, as predicted by PBPK studies [29,30]. Unbound efavirenz concentrations are not available in the published studies. However, Lamorde et al. hypothesised that the 25% decrease in efavirenz’s Ctrough measured during the third trimester versus postpartum was due to a change in protein binding caused by a decrease in albumin during the late stages of pregnancy (31%), leading to a higher unbound fraction of efavirenz, which would result in lower total drug concentration measurements [28].

**Figure 2:**
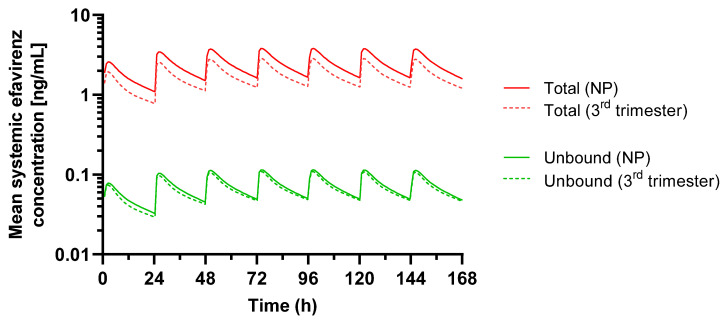
Simulated concentrations of total (red) and unbound (green) efavirenz in a nonpregnant population (NP; solid lines) and during the 3rd trimester of pregnancy (dashed lines). A dosing regimen of 600 mg of efavirenz per day for seven days was used.

A pregnancy PBPK model is available in the SIMCYP V21 and GASTROPLUS 9.8.2 [31,32]. This PBPK model was used to simulate the unbound concentrations of efavirenz in both nonpregnant and pregnant populations (Table 1). The simulation results in nonpregnant populations versus those in the third trimester, which is considered to represent the biggest change, are reported in Figure 2.

Changes in efavirenz’s PK during pregnancy resulted in a predicted decreased exposure in pregnancy of the total drug by 27%. Blood binding and partitioning into cells is low for this drug, therefore, the predicted blood concentrations are lower than those in the plasma. The plasma protein binding is high, and the pregnancy-related changes in the plasma protein binding contribute to the decrease in exposure of the total drug, and a significantly smaller decrease in the exposure to the free, pharmacologically active efavirenz (7%) was seen.

## 3. Tacrolimus

Tacrolimus is indicated for the prophylaxis of transplant rejection in adult kidney or liver allograft recipients, as well as for treatment of allograft rejection resistant to other immunosuppressive medicinal products in adult patients [15].

Due to medical needs, pregnant women can be treated with tacrolimus when there is no safer alternative and when the perceived benefit justifies the potential risk to the foetus [15]. Tacrolimus shows extensive blood partitioning and is highly bound (>98.8%) to plasma proteins, mainly serum albumin and AAG, and it is almost completely metabolized by the CYP3A4 [15]. A number of authors investigated the effect of pregnancy on the PK of tacrolimus [7,8,13,33,34,35,36]. Zheng et al. observed decreased total blood tacrolimus concentrations in pregnancy, whereas no significant change in unbound concentrations were measured [7].

In order to maintain tacrolimus’ trough blood concentrations in the therapeutic range, in the Zheng study [7], tacrolimus doses were increased by approximately 45% in pregnancy. However, tacrolimus’ free fraction increased by 91% in plasma and by 100% in blood during pregnancy, while the serum albumin’s concentrations decreased by 27%. The increased dose led to increased unbound tacrolimus’ trough concentrations and AUC, 112% and 253%, respectively, in pregnancy compared to the nonpregnant population. Similarly, the whole blood CL/F was 39% higher in pregnancy than in postpartum (47.4 ± 12.6 vs. 34.2 ± 14.8 L/h), while no statistically significant difference was observed in unbound CL/F in pregnancy compared to postpartum.

**Figure 3:**
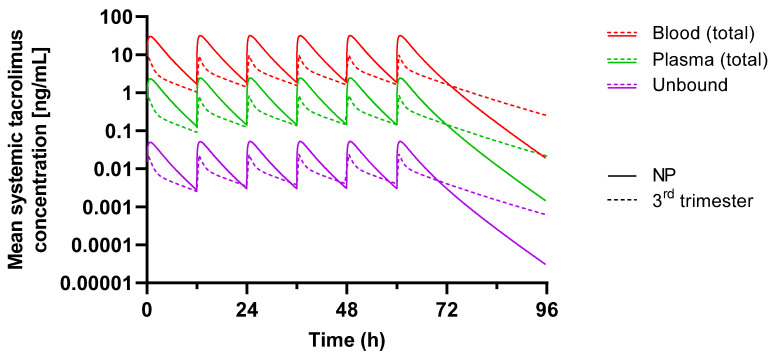
Simulated concentrations of total tacrolimus in blood (red) and plasma (green) and of unbound tacrolimus levels (purple) in a nonpregnant population (NP; solid lines) and during the 3rd trimester of pregnancy (dashed lines). In order to match the observed patient data (see Table 1), a dosing regimen of 2.8 mg every 12 h was used for the nonpregnant population, and a dosing regimen of 4.05 mg every 12 h was used for the pregnant population.

**Figure 4:**
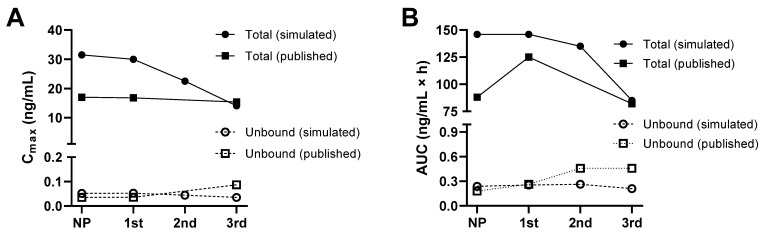
Summary of simulated and published changes in whole blood levels of total and unbound tacrolimus during pregnancy. Changes in Cmax (**A**) and AUC (**B**) are presented. Published data are taken from Zheng et al., 2012 [7]. Note that the published data shown within the 3rd trimester represent a mean of samples taken from both 22–26 weeks and 34–38 weeks.

A PBPK model was previously developed and used to simulate the total and unbound systemic plasma and blood exposure of tacrolimus in nonpregnant and pregnant populations during the first, second, and third trimester [19]. The observed and simulated clinical PK data for tacrolimus are presented in Table 2. Figure 3 shows the simulated tacrolimus (total and unbound) plasma and blood in the pregnant, third trimester, and nonpregnant population, and Figure 4 shows the changes over the different trimesters. Unbound concentrations represent both blood and plasma, as these are expected to be at equilibrium.

Tacrolimus extensively binds to erythrocytes; therefore, blood concentrations are predicted to be much higher than plasma concentrations in both nonpregnant and pregnant subjects; protein binding is high and, therefore, unbound concentrations are much lower. The model predicted a 60% decrease in the total blood tacrolimus’ AUC, compared to a lower, 40% decrease in the unbound concentrations in the third trimester compared to nonpregnant women.

## 4. Discussion

### 4.1. Efavirenz

In the efavirenz’s PBPK model, changes in the efavirenz’s PK during pregnancy resulted in a predicted, decreased exposure in pregnancy of the total drug by 27%, which was consistent with the observed data. The model also allowed for an understanding of how the concentrations in different matrixes (e.g., plasma versus blood) or components (e.g., total versus free drug) are altered in pregnancy based on known physiological changes. Blood binding and partitioning into cells is low for this drug; therefore, the predicted blood concentrations are lower than those in plasma. The plasma protein binding is high; various figures are reported in the literature, and the model uses the lowest bound value of 97%, which appears to best fit the clinical data [31,37]. This figure is also supported by the data on HIV-1-infected patients (n = 9) who received 200 to 600 mg of efavirenz once daily for at least one month and in whom the cerebrospinal fluid concentrations (which can be considered to represent the free drug) ranged from 0.26 to 1.19% (mean 0.69%) of the corresponding plasma concentration. It should be considered that disease states such as HIV-infection can also moderate the levels of plasma protein and that this can also be included in the PBPK model [38]. The free concentrations in plasma and blood are much lower than the total concentrations in both nonpregnant and pregnant subjects, but, due to the reduced albumin concentrations in pregnancy, the difference is less, and, thus, it can be seen that the change in the unbound efavirenz’s ratio is reduced to only 7%. This could be explained by the clearance unbound, which is expected to remain constant due to physiological compensation mechanisms.

It has been suggested that the lower total exposure of efavirenz could still be sufficient for efficacy; however, this has been questioned as the modelling shows that some subjects may not reach the minimum effective total concentration (MEC) of 1 mg/L required for viral suppression. In addition, lower doses have been proposed in pregnancy based on the clinical study ENCORE1, which demonstrated the non-inferiority of daily 400 mg of efavirenz versus 600 mg over 96 weeks in treatment-naive, HIV-infected adults [26]. This dose may not be supported based on the changes in total concentrations; however, with the consideration of the free concentrations, with only an expected 7% change in concentration, full efficacy would be expected and such a reduced dose could be considered. It should also be noted that the concentrations are likely to be dependent on the CYP2B6 genotype as the enzymes involved in the clearance are polymorphic. However, while pregnancy-related changes in CYP3A4 are captured in the model, CYP2B6 activity in the model does not change likely due to insufficient available data to inform the model.

### 4.2. Tacrolimus

Tacrolimus extensively binds to erythrocytes, but it does not bind to haemoglobin; instead, an intracellular protein in erythrocytes, corresponding to FKBP, is suggested to be responsible [39]. Blood concentrations are therefore much higher than plasma concentrations in both nonpregnant and pregnant subjects. The PBPK model demonstrates this difference. Plasma protein binding is also high so that free concentrations in plasma and blood are much lower. Based on the doses described in Zheng’s study [7], the model predicted a 40% and 60% decrease in the unbound and total blood tacrolimus’ AUC, respectively, in the third trimester compared to nonpregnant women. Calculation of the PK’s parameters using the published tacrolimus concentrations data [7] shows a 75% increase and a 36% decrease in the unbound and total tacrolimus’ AUC, respectively, in the third trimester compared to nonpregnant women (Figure 4). Although the model shows a reduced effect on the free drug, this is not as pronounced as it is in the clinical data, where an increase in free drug concentrations were seen. The mis-prediction may be due to changes in the enzymes and transporters involved in the disposition and clearance of the drug not being fully characterised in the current model. Irrespective of this, the model agrees with the clinical data in demonstrating the free tacrolimus levels to change less than the total tacrolimus levels.

Changes in the tacrolimus’ PK during pregnancy result in an increased unbound-to-whole-blood tacrolimus concentration ratio. Some authors have discussed the need to increase the dose and intensify therapeutic drug monitoring to maintain tacrolimus’ total concentrations within the target range [33]. However, the same authors suggested that a dose adjustment might not be necessary in pregnancy because of the higher unbound concentrations. The modelling partially supports this. In addition, tacrolimus’ whole blood CL/F is inversely correlated with both the haematocrit and red blood cell counts, suggesting that the binding of tacrolimus to erythrocytes restricts its availability for the metabolism. Further complexities may occur in hypoalbuminemic and/or anaemic patients, which could be attributed to the disease state, as unbound concentrations might be considerably higher than those measured in nonpregnant populations [7,33]. Moreover, changes in lipoprotein concentrations may potentially affect the percentage of tacrolimus content in lipoprotein fractions, leading to a possible variation in tacrolimus’ unbound fraction. [40,41,42] Based on the available data and considering the unbound concentration, no dosage adjustments may be required in pregnancy.

### 4.3. Methodology to Determine the Exposure Changes

The effect of pregnancy-related changes (e.g., altered activity of metabolic Phase I and Phase II enzymes) on the PK of drugs is usually evaluated on a comparison of total systemic exposure in pregnancy versus nonpregnant subjects. In some cases, this may be used to support dosing adjustments. However, free drug concentrations are the ones of importance for cellular diffusion and cellular distribution and are usually considered to be the pharmacologically active form. The concentration of drug-binding proteins within the blood decreases during pregnancy, which alters the unbound plasma concentrations of drugs, particularly those that are highly protein-bound. For drugs with a low extraction ratio, only the unbound drug penetrates membranes and is available for elimination; an increase in the unbound fraction will, therefore, proportionally increase the clearance. For some medicinal products, even a minor change in the level of protein binding might influence its efficacy [2,38]. The Medicine and Healthcare products Regulatory Agency (MHRA) in the United Kingdom recently published a systematic review which highlights the fact that total and free concentrations data are not always measured in clinical studies or reported in the literature and that this can limit the evaluation of the impact of pregnancy on the unbound concentrations which are relevant to therapeutic effectiveness [43].

When faced with a change in total drug, it is important to consider free drug exposures. Different changes in concentrations of the free drug than those of the total drug may be clinically significant and have important implications for dose adjustment for highly bound medicines such as tacrolimus, efavirenz, clindamycin, phenytoin, and carbamazepine.

Whilst measurement of unbound drug levels is therefore desirable, bioanalytical challenges arise when measuring free concentrations, as reliable and highly sensitive methods are required to separate total and free drug fractions and detect small quantities of unbound drug. Musteata et al. indicated some of the bioanalytical challenges for measuring free drugs, which included high resource-consuming methodologies, e.g., separation techniques used in some methods of measurement of free drug, such as equilibration procedures which can take days [44], and a higher variability in the measured free concentrations due to several sample processing steps [44]. In addition, some physiochemical characteristics of the drugs, such as a low partition coefficient in nonpolar phases, may lead to difficulties in separating the free fraction [44]. Also, extreme lipophilicity as in case of ciclosporin and tacrolimus may necessitate the use of stainless-steel equipment to prevent the drug’s adherence to the plastic walls of the equilibrium dialysis equipment [45].

In addition to these technical challenges, the measurement of free and total drug levels in a clinic can be time consuming due to the need to organise patient visits, sampling, and sample processing. Turn-around time of samples is often in the region of one to two days, but other delays occur in the shipment of samples and communications which can lead to adjustments being made based on outdated information.

### 4.4. Application to Other Medicines of Interest

A number of other highly bound compounds exhibit a reduction in the total level of drug during pregnancy, but with the levels of free drug not changing significantly (e.g., carbamazepine and phenytoin [46,47,48]) or not being reported (e.g., lumefantrine, dolutegravir, and clindamycin [49,50]). For drugs in which the free level has not been investigated, PBPK modelling provides an opportunity to predict free blood concentrations and, therefore, to begin assessing the implications for clinical safety and efficacy. Such studies may also further highlight the issues of only using total drug levels to inform dose adjustment.

This can be exemplified by lumefantrine, for which free concentrations during pregnancy are not available in the literature. At present, dosing adjustment in pregnant population appears to rely on the total drug level, which could be deceptive, as described above.

Lumefantrine is a CYP3A4 substrate lumefantrine, which exhibits high protein binding (99.7%) [51]. This medicine can be used in combination with artemether during the second and third trimester of pregnancy if the expected benefit to the mother outweighs the risk to the foetus [51]. The World Health Organization (WHO)’s label states that the above combination can be used during pregnancy [52]. Some authors suggest that the increased HDL levels would result in a decrease in the volume of distribution and in the raising of the total plasma lumefantrine concentrations [53]. However, in a number of clinical studies, a >30% decrease in the total plasma lumefantrine concentrations has been observed in the pregnant population compared to the nonpregnant group, consistent with the expected increase in CYP3A4 activity [53,54]. Using population PK modelling, Onyamboko et al. [55] also predicted that 38% of the study’s population had day 7 lumefantrine venous blood concentrations below the target concentration threshold of <280 ng/mL. Similarly, the population PK analysis [56,57] predicted a ~30% decrease in lumefantrine exposure during pregnancy. Due to lumefantrine’s dose-saturable absorption, the WHO recommends extending the treatment duration from a 3- to a 5-day regimen to increase the total systemic exposure of lumefantrine in the pregnant population [52].

### 4.5. PBPK Models—Opportunities and Limitations

PBPK modelling offers an opportunity to predict the free drug concentrations for highly bound drugs, taking into considerations the different physiochemical properties of the studied drugs. PBPK modelling presents a rapid, reproducible alternative to existing methodologies that may be used to support dosing strategies, as is shown above for both efavirenz and tacrolimus, and can, therefore, be employed to predict whether changes in dose regimen are sufficient and/or necessary. Lumefantrine (discussed above), however, is highly bound to HDL, not albumin or AAG. Pregnancy-related changes in HDL are not currently incorporated within the PBPK models, but data are available [58] and so studies could be performed when these data are integrated into the models.

Whilst pregnancy PBPK models serve as a useful tool to support dosing strategies, a number of limitations are currently associated with the methodology. For example, there are other changes that occur in plasma composition during pregnancy that are not fully incorporated in the models. Ghio et al. reported that, from the 12th week of gestation, phospholipids, cholesterol (total, LDL, HDL), and triglycerides (TG) increase in response to oestrogen stimulation and insulin resistance [59]. Changes in HDL levels may have an effect on the tissue distribution and free systemic exposure of medicines highly bound to that protein. At present, changes in these compounds are not incorporated into the models and so, for drugs which highly bind to these (e.g., lumefantrine [53]), the pregnancy PBPK models are unlikely to accurately predict changes in exposure to free drug.

Similar limitations apply to the incorporation of changes in enzyme activity. Whilst the SIMCYP pregnancy modules includes changes in some CYP enzymes during gestation (e.g., CYP1A2, CYP2D6, and CYP3A4) no change in the activity of others (e.g., CYP2B6 [efavirenz], CYP2C9 [phenytoin], CYP3A5 [clindamycin], and UGT2B7 [carbamazepine]) is included, typically due to a lack of in vitro data to support the models. Further details of what is currently incorporated in the model can be found in previous publications [17,18,60]. As such, the continued collection and incorporation of data are necessary to support the advancement of the PBPK models.

There are other intrinsic and extrinsic factors that can impact the exposure of medicines in both non-pregnant and pregnant subjects, and these should be considered. Pregnant patients may need multi-therapy treatments; therefore, the potential risk of DDI between co-medications needs to be considered. PBPK can be used to support this evaluation; however, the interaction mechanism should be sufficiently accounted for in the pregnancy model.

Other possible factors are related to real-life parameters. Population characteristics, e.g., age, body weight, age, BMI, and fluids/food intake, can impact exposure and should ideally be incorporated into the model, and the effect of these variables on the predicted PK profiles in pregnancy should be considered. The PBPK models allow studies to be run on virtual populations in which physiological parameters differ between ‘participants’ and, thus, such variability can be accounted for. In addition to recapitulating natural variation, this also allows models to be created in a way which reflects best- and worst-case scenarios to investigate the effects of specific parameters on drug concentrations. However, again, there are some gaps in the knowledge of physiology associated with some of these parameters, and the confidence in the model should be considered on a case-by-case basis when supporting dosing decisions.

Pregnancy-related physiological changes are accounted for in the mostly used PBPK platforms; however, differences across modelling systems have not been evaluated in this work.

The pregnancy PBPK models offer a good opportunity to study the effect of physiological changes in pregnancy and are actively being developed with additional changes, such as the inclusion of additional proteins and enzymes. As the models develop, their potential to allow for the study of the effect of the many physiological changes in pregnancy on the true exposure of medicines in these subjects holds great potential.

## 5. Conclusions

The effect of pregnancy-related changes on plasma proteins concentrations and blood partitioning may lead to differential changes in free concentrations compared to total concentrations of drugs. Free concentrations are generally considered to be the pharmacologically active form and are important for cellular diffusion and distribution. Despite this, both total and free concentrations data are not always evaluated in clinical studies during pregnancy, causing uncertainty regarding conclusions of efficacy doses in this population. The PBPK models may be a useful tool to investigate the changes in plasma proteins binding and simulation of free and total concentrations in pregnancy to inform dosing adjustment in such a population. The models are currently limited by the lack of incorporation of changes in some plasma components, e.g., lipoproteins, enzymes such as UGTs, and transporters. These data are necessary to support the advancement of the PBPK models. As the models develop, their potential to allow for the study of the effect of the many physiological changes in pregnancy on the true exposure to drugs in these subjects holds great potential.

## Figures and Tables

**Table 1:** Simulated and published PK parameters of efavirenz in pregnant and nonpregnant population.

Compound and Dosing Regime	Measured Parameter	Pregnancy Period	PK Parameters				
			Simulated Data			Published Data	
			Cmax (mg/L)	AUC (mg/L·h)	AUC (P)/AUC (NP)	Cmax (mg/L)	AUC (mg/L·h)
Efavirenz 600 mg/Day 7 days	Plasma (Total)	NP	3.75	60.2		4.41 ^a^ [23] 5.10 ^b^ [25] 3.93 ^c^ [24]	62.7 ^a^ [23] 58.3 ^b^ [25] 62.6 ^c^ [24]
		1st	3.4	53.8	89%	-	-
		2nd	3.1	49.4	82%	3.87 ^a^ [23]	47.3 ^a^ [23]
		3rd	2.8	44.1	73%	5.13 ^a^ [23] 5.44 ^b^ [25] 4.33 ^c^ [24]	60.2 ^a^ [23] 55.4 ^b^ [25] 52.3 ^c^ [24]
	Plasma (Unbound)	NP	0.13	1.81			
		1st	0.11	1.71	94%	-	-
		2nd	0.11	1.71	94%	-	-
		3rd	0.11	1.69	93%	-	-
	Blood (Total)	NP	2.92	46.6			
		1st	2.7	42.2	91%	-	-
		2nd	2.5	39.1	84%	-	-
		3rd	2.25	35.43	76%	-	-
	Blood (Unbound)	NP	0.13	1.81			
		1st	0.11	1.71	94%	-	-
		2nd	0.11	1.7	94%	-	-
		3rd	0.11	1.69	93%	-	-

^a^ Kreitchmann at al [23] (median). Non-pregnant values were measured in the study cohort 6–12 weeks postpartum. ^b^ Cressey et al. [25] (median). Non-pregnant values were measured in the study cohort 6–12 weeks postpartum. ^c^ Lartey et al. [24] (geometric mean). Non-pregnant values were measured in the study cohort 6 weeks postpartum. AUC = area under the plasma drug concentration–time curve; Cmax = maximum drug concentration; P = pregnancy; and NP = nonpregnant.

**Table 2:** Simulated and published PK parameters of tacrolimus in pregnant and nonpregnant populations.

Compound, Measured Parameter	Dose (mg/Day)	Pregnancy Period	PK Parameters				
			Simulated Data			Published Data	
			Cmax (ng/mL)	AUC (ng/mL·h)	AUC (P)/AUC (NP)	Cmax (ng/mL)	AUC (ng/mL·h)
Tacrolimus, blood (Total)	5.6	NP	31.5	146		17.0 ^a^ [7]	88.1 ^a^ [7]
	6.9	1st	30.0	146	100%	16.8 ^a^ [7]	125.0 ^a^ [7]
	8.1	2nd	22.5	135	92%	15.4 ^a^ [7]	82.0 ^a^ [7]
	8.1	3rd	14.1	85	58%		
Tacrolimus, blood (Unbound)	5.6	NP	0.052	0.239		0.035 ^a^ [7]	0.181 ^a^ [7]
	6.9	1st	0.053	0.254	106%	0.036 ^a^ [7]	0.263 ^a^ [7]
	8.1	2nd	0.044	0.263	110%	0.087 ^a^ [7]	0.458 ^a^ [7]
	8.1	3rd	0.035	0.209	87%		
Tacrolimus, plasma (Total)	5.6	NP	2.43	11.3		-	-
	6.9	1st	2.36	11.4	101%	-	-
	8.1	2nd	1.83	10.9	96%	-	-
	8.1	3rd	1.24	7.44	66%	-	-

^a^ Calculated using the mean PK time-profiles presented by Zheng et al. [7]. Note that this study pooled data from the second (22–26 weeks gestation) and third (34–38 weeks gestation) trimesters. Nonpregnant values were taken from the study’s cohort >3 months postpartum. AUC = area under the plasma drug concentration–time curve; Cmax = maximum drug concentration; NP = nonpregnant; and P = pregnancy.

## Data Availability

Additional data are available upon request to the authors at the email address susan.cole@mhra.gov.uk.
